# Comparison of Overhauser DNP at 0.34 and 3.4 T with Frémy’s Salt

**DOI:** 10.1007/s00723-012-0362-5

**Published:** 2012-06-03

**Authors:** M.-T. Türke, M. Bennati

**Affiliations:** Max Planck Institute for Biophysical Chemistry, 37077 Göttingen, Germany

## Abstract

Dynamic nuclear polarization (DNP) is investigated in the liquid state using a model system of Frémy’s salt dissolved in water. Nuclear magnetic resonance signal enhancements at 0.34 and 3.4 T of the bulk water protons are recorded as a function of the irradiation time and the polarizer concentration. The build-up rates are consistent with the *T*_1n_ of the observed water protons at room temperature (for 9 GHz/0.34 T) and for about 50 ± 10 °C at 94 GHz/3.4 T. At 94 GHz/3.4 T, we observe in our setup a maximal enhancement of −50 at 25 mM polarizer concentration. The use of Frémy’s salt allows the determination of the saturation factors at 94 GHz by pulsed ELDOR experiments. The results are well consistent with the Overhauser DNP mechanism and indicate that higher enhancements at this intermediate frequency require higher sample temperatures.

## Introduction

Dynamic nuclear polarization (DNP) of the Overhauser type (OE-DNP) [1, 2] can provide a valuable tool to enhance the sensitivity of nuclear magnetic resonance (NMR) experiments in the liquid state [3–5].

A polarizer molecule carrying an unpaired electron spin (mostly a stable radical like a nitroxide) is inserted into the liquid-state NMR sample under investigation. By continuous wave microwave irradiation on the EPR transition of the unpaired electron a steady-state polarization (saturation) of this transition is achieved, which in turn polarizes coupled NMR transitions via cross relaxation pathways.

In recent years, detailed mechanistic studies have provided insight into the specific parameters needed to achieve optimal enhancements of liquid-state NMR signals, such as polarizer type, polarizer concentration, microwave power or the magnetic field at which the polarization step is carried out [6–11]. Moreover, high DNP enhancements of water proton signals up to two orders of magnitudes have been recently reported not only at low microwave frequencies (9 GHz, [7–9]) but also at intermediate (94 GHz, [12, 13]) and high (260 GHz, [14]) microwave frequencies. The enhancements at 94 GHz/3.4 T in [12, 13] were much higher than in our in initial reports [6, 7]. Irradiation times needed to reach maximum DNP effects have mainly been optimized experimentally, and the build-up of dynamic nuclear polarization has been interpreted with the nuclear relaxation time *T*_1n_ and its temperature dependence [12, 13]. In order to rationalize the observed frequency dependence of liquid DNP enhancements up to medium frequency/fields and particularly compare the maximal enhancements reported so far at 94 GHz in our [7] and in other groups [12, 13], we have measured the DNP signals as a function of the irradiation time and compared with independent *T*_1n_ measurements [8, 15]. Furthermore, we have determined the saturation factors for ^15^N-labelled Frémy’s salt at 94 GHz via pulsed electron double resonance (ELDOR) experiments [16]. The results allow us to rationalize quantitatively our enhancements at 94 GHz in terms of the Overhauser mechanism.

## Overhauser DNP in Liquids

In Overhauser DNP, the enhancement of the NMR signal *ε* is derived from the steady-state solution, i.e. ${\text{d}}\left\langle {I_{\text{z}} } \right\rangle /{\text{d}}t = 0$, of the Solomon equations [17], where the nuclear polarization is described as:

1 $$ \frac{{{\text{d}}\left\langle {I_{z} } \right\rangle (t)}}{{{\text{d}}t}} = - (\rho + w^{0} )\left( {\left\langle {I_{z} } \right\rangle (t) - I_{0} } \right) - \sigma \left( {\left\langle {S_{z} } \right\rangle (t) - S_{0} } \right) $$

Here, $\left\langle {I_{\text{z}} } \right\rangle {\text{ and }}\left\langle {S_{\text{z}} } \right\rangle$ are the expectation values of the nuclear and electron magnetizations, respectively, whereas *I*_0_ and *S*_0_ are the corresponding values at thermal equilibrium. $\rho = w_{0} + 2w_{ 1} + w_{2}$ and $\sigma = w_{2} - w_{0}$ are parameters containing the relaxation rates of the transitions within the four-level system of two coupled spins $S = I = {1 \mathord{\left/ {\vphantom {1 2}} \right. \kern-\nulldelimiterspace} 2}$, i.e. the nuclear single-quantum relaxation rate *w*_1_, as well as the zero- and double-quantum cross-relaxation rates, *w*_0_ and *w*_2_. *w* ^0^ is the nuclear relaxation rate in the absence of paramagnetic molecules and accounts for nuclear relaxation not induced by the electron.

Although a similar equation as Eq. (1) can be set up for the electron spins, such a description would not be meaningful as they are subject to much faster relaxation pathways [2]. In the simple case of a single homogeneous EPR line, the expectation value $\left\langle {S_{\text{z}} } \right\rangle$ can be extracted from the Bloch equations [18]. For a more complicated system, e.g. a nitroxide radical exhibiting two or three EPR lines, an explicit treatment of the equation of motion of the spin density matrix is necessary [8, 19].

Notwithstanding, in the most general case, the electronic magnetization can be considered in the form of an overall saturation factor $s = \left( {S_{0} - \left\langle {S_{z} } \right\rangle } \right)/S_{0}$, which describes the extent to which the electronic magnetization is driven away from thermal equilibrium by microwave irradiation.

The steady-state solution of Eq. (1) then directly yields the Overhauser equation:

2 $$ \varepsilon = \frac{{\left\langle {I_{\text{z}} } \right\rangle }}{{I_{0} }} = 1 - \xi fs\frac{{\left| {\gamma_{\text{S}} } \right|}}{{\gamma_{\text{I}} }} $$

with coupling factor $\xi = \sigma /\rho$, leakage factor $f = \rho /(\rho + w^{0} )$ and the ratio of the respective gyromagnetic ratios of the electron and the nucleus, *γ*_S_ and *γ*_I_.

In order to describe the kinetics of the DNP build-up, we consider the time-dependent solution of Eq. (1). It is important to note that two different time-scales describe the formation of electron polarization (saturation) and dynamic nuclear polarization. While the former is reached within several hundred ns to μs for conventional liquid state DNP polarizers (e.g. nitroxide radicals) in water given by the electron longitudinal relaxation time *T*_1e_, the latter is observed to be in a range of several seconds. Hence, assuming that $\left\langle {S_{\text{z}} } \right\rangle - S_{0}$ is constant on the time-scale of DNP build-up and given by −*sS*_0_, we can neglect the time dependence of the electron magnetization, and rewrite Eq. (1) as a single inhomogeneous differential equation:

3 $$ \frac{{{\text{d}}\left\langle {I_{z} } \right\rangle (t)}}{{\text{d}}t} + (\rho + w^{0} )\left( {\left\langle {I_{z} } \right\rangle (t) - I_{0} } \right) = \sigma sS_{0} $$

With the initial condition, nuclear polarization starts from the thermal equilibrium value, i.e. $\left\langle {I_{\text{z}} } \right\rangle (0) = I_{0}$, the solution of Eq. (3) results to:

4 $$ \left\langle {I_{z} } \right\rangle (t) = I_{0} - \frac{{\sigma sS_{0} }}{{\rho + w^{0} }}\left( {e^{{ - (\rho + w^{0} )t}} - 1} \right). $$

Rearrangement in a similar way as Eq. (2) yields a ‘time-dependent Overhauser equation’ that describes the build-up of nuclear polarization with duration of microwave irradiation for *t* > > *T*_1e_:

5 $$ \varepsilon (t) = \frac{{\left\langle {I_{z} } \right\rangle (t)}}{{I_{0} }} = 1 - \xi fs\frac{{\left| {\gamma_{S} } \right|}}{{\gamma_{I} }}\left( {1 - e^{{ - t/T_{1n} }} } \right). $$

Accordingly, the time constant of DNP build-up is given by the observable nuclear relaxation rate, i.e. $1/T_{{ 1 {\text{n}}}} = \rho + w^{0} = w_{0} + 2w_{1} + w_{2} + w^{0} .$

## Experimental

^15^N-labelled Frémy’s salt was synthesized in house and dissolved in 50 mM K_2_CO_3_ buffer (pH ≈ 11) to produce 0.5, 5, 10, 25 mM solutions of the disulfonate anion ON(SO_3_^−^)_2_. Concentrations were checked by optical absorption [20]. Samples were degassed for 10 min by N_2_ flow. For experiments at 9.6 GHz/0.34 T, they were loaded into 0.45 mm inner diameter (ID) tubes to a height of 3 mm and sealed; at 94 GHz/3.4 T, they were filled into 0.1 mm ID capillaries to a height of 5 mm and sealed. The effective sample volumes were kept as small as possible to minimize the heating effects and amounted to 0.5 μL at 9 GHz/0.34 T and 40 nL at 94 GHz/3.4 T. For the inversion recovery experiment at 94 GHz/3.4 T, we have used a larger sample tube of 0.5 mm ID filled to 10 mm height (volume: 2 μL) to achieve higher NMR signal intensities.

The DNP experiments were performed using the X-band and W-band DNP spectrometers (0.34 T/3.4 T, 9.6 GHz/94 GHz EPR, 15 MHz/140 MHz ^1^H NMR) specified earlier [7] with a new custom-built NMR console (Bruker GmbH) for 140 MHz NMR experiments. At 9.6 GHz a Bruker dielectric ENDOR resonator and at 94 GHz a Bruker cylindrical ENDOR resonator operating in TE_011_ mode were employed.

For all DNP measurements, maximum microwave power corresponding to *B*_1_ ≈ 4–5 G (9.6 GHz) or 2–3 G (94 GHz) was applied [7]. The DNP enhancements were quantified by comparison of the NMR signal after microwave irradiation to the one at thermal equilibrium. 1,024–4,096 scans were averaged at thermal equilibrium, while only 8–32 scans were needed to achieve sufficient data quality for the DNP-enhanced NMR signals.

Pulsed ELDOR experiments were performed as reported earlier [16] using a home-built dual mode 94 GHz resonator [21]. A saturation pulse of 1 μs length was applied at various frequencies prior to EPR FID detection with a 40 ns pulse on resonance with one of the two ^15^N hyperfine lines. The modes of the dual mode resonator were adjusted, so that a similar *B*_1_ of up to 2 G is reached at the position of the two EPR lines.

## Results and Discussion

### DNP Build-up at 9.6 GHz/0.34 T

**Fig. 1:**
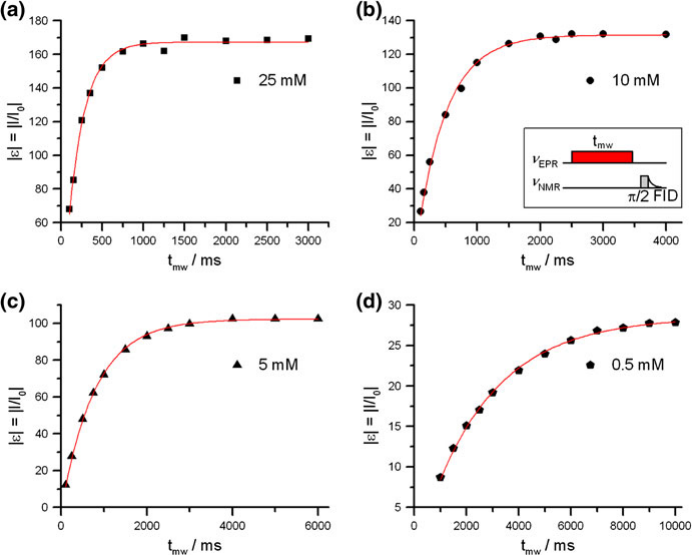
Enhancement *ε* of ^1^H NMR signals of bulk water as a function of microwave irradiation time *t*_mw_ for different Frémy’s salt concentrations at 0.34 T fitted according to Eq. (5). The *inset* shows the pulse sequence applied in all DNP experiments

In Fig. 1, DNP enhancements of the bulk water ^1^H are plotted as a function of irradiation time for four different concentrations of polarizer. Clearly, the build-up proceeds faster if the radical concentration is higher. Maximum enhancements are reached at around 1, 2, 4 and 10 s for 25, 10, 5 and 0.5 mM samples, respectively, and amount to −170, −130, −100 and −30 (within an error of 10 % or less) in agreement with our former observations [8] (Fig. 1).

Fitting with Eq. (5) shows that all curves are described very well by a monoexponential function with time constants 2,590 ± 40 ms, 800 ± 20 ms, 470 ± 20 ms, and 200 ± 10 ms at 0.5, 5, 10, and 25 mM polarizer concentrations, respectively.

Table 1 compares these values to the ^1^H nuclear relaxation times *T*_1n_ extracted from nuclear magnetic relaxation dispersion measurements (NMRD), which have been reported earlier [8]. Underlying the *T*_1n_ times is the relaxivity *R* determined with the 5 mM sample. The *T*_1n_ at radical concentration *c* is obtained as

6 $$ \frac{1}{{T_{{ 1 {\text{n}}}} }} = R \cdot c + R_{\text{dia}} $$

with *R*_dia_ being the diamagnetic relaxation rate in the absence of the polarizer.

**Table 1:** Comparison of the characteristic DNP build-up times *T*_buildup_ at 0.34 T in liquid solution to the nuclear relaxation times *T*_1n_ extracted from NMRD [8] at room temperature

Polarizer concentration *c*/mM	*T*_buildup_/ms	*T*_1n_ (NMRD, 298 K)/ms
0.5	2,590 ± 40	2,420
5	800 ± 20	800
10	470 ± 20	460
25	200 ± 10	200

A measurement of *T*_1n_ using the DNP setup at 9.6 GHz is less accurate due to the weakness of the NMR signal in the absence of DNP. Furthermore, an alternative measurement of *T*_1n_ via decay of the DNP signal would not allow a priori for a definite temperature in the sample.

The *T*_1n_ data in Table 1 show best agreement with the observed build-up time constant, i.e. *T*_1n_ = 200 ms at 25 mM, *T*_1n_ = 460 at 10 mM and *T*_1n_ = 800 at 5 mM. At the lowest concentration discussed here, i.e. at 0.5 mM, the observed nuclear relaxation times are slightly smaller than the time constant describing DNP build-up with irradiation time. However, we note that the error might be large at this concentration as the experimental window of irradiation times does not yield a full characterization of the exponential build-up curve with very long time constant (see Fig. 1). We conclude that the build-up of the DNP signal is best described by the *T*_1n_ of the polarized nuclei at room temperature and confirm that our reported maximal enhancements at 9.6 GHz are not affected by temperature effects [7].

### Experiments at 94 GHz/3.4 T

#### DNP Build-up

**Fig. 2:**
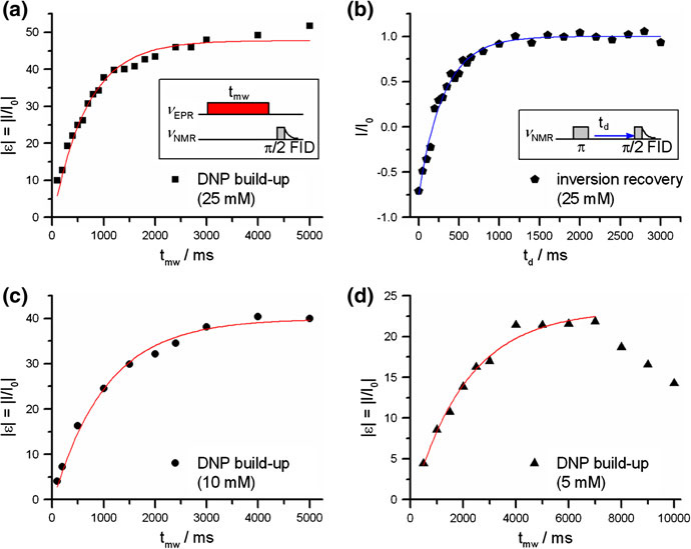
**a**, **c**, **d** Enhancement *ε* of ^1^H NMR signals of bulk water containing ^15^N-labelled Frémy’s salt at 3.4 T as a function of microwave irradiation time *t*_mw_ fitted according to Eq. (5). *Inset*: pulse sequence applied in the DNP experiments. In **d** we observe a decomposition of the sample at irradiation times longer than 7 s (Frémy’s salt is known to decompose in solution [22]. The rate of decomposition depends on the pH value and the temperature. We observe decomposition only at long irradiation times (up to 10 s), which were only applied for the 5 mM sample). **b** ^1^H NMR inversion recovery experiment on a 25 mM sample at 3.4 T

To compare the performance at 3.4 T, we have measured the enhancements as a function of irradiation time for 5, 10 and 25 mM ^15^N-labelled Frémy’s salt. The maximum measured enhancements at these concentrations amount to −22, −40 and −52 using 7, 5 and 5 s of microwave irradiation, respectively (Fig. 2). The error is estimated to be around 20 % due to the very low intensity of the thermal equilibrium signal. However, this error should not affect the build-up time constants since each time point is divided by the thermal equilibrium signal in the same fashion. As expected from the experiments at 0.34 T, where 25 mM ^15^N-Frémy’s salt and TEMPONE-D,^15^N have yielded similar maximum enhancements [8], the enhancement of ε = −52 observed here is close to the one recorded with 25 mM TEMPONE-D,^15^N in an earlier experiment, i.e. ε = −43 [7].

However, these enhancements are considerably lower than the recently reported values of up to −170 using TEMPOL at 3.4 T [12, 13]. In both reports from the literature, these high enhancements were attributed to elevated sample temperatures during microwave irradiation exceeding 100 °C.

For a better comparison of our data with the literature, we have fitted the DNP build-up curves up to 5 s at 25, 10 mM and up to 7 s at 5 mM polarizer concentration using Eq. (5) and obtained time constants of 670, 1,030 and 2,060 ms, respectively (Table 2). These values are 1.3–1.9 times longer than the respective *T*_1n_ (298 K) resulting from NMRD with a relaxivity of 0.0998 s^−1^ mM^−1^ [8] and Eq. (6). For a control, the room temperature relaxation time at 25 mM was directly measured in an inversion recovery experiment (Fig. 2b) providing *T*_1n_ (298 K) = 310 ms in satisfactory agreement with the NMRD data [*T*_1n_ (298 K, 25 mM) = 360 ms].

**Table 2:** Comparison of Overhauser DNP parameters at 3.4 T and average temperature of 323 K

Conc. *c*/mM	*ε*_max_	*T*_buildup_/ms	*f*	*s*_eff_ (ELDOR)	*ξ* [calc. from Eq. (2)]	*ξ* ^a^ (NMRD, Ref. [15])
5	−22	2,060 ± 150	0.63	0.51	0.11	0.11/0.14
10	−40	1,030 ± 80	0.78	0.66	0.12	0.11/0.14
25	−52	670 ± 40	0.90	0.80	0.11	0.11/0.14

^a^Derived for TEMPONE-D,^15^N in water without inclusion of contact contribution for temperatures 318/328 K

The observed build-up times suggest that some heating is occurring in our 3.4 T DNP setup as opposed to the DNP experiment at 0.34 T. To estimate the temperature underlying the *T*_1n_ and the *T*_buildup_ values, we have simulated the temperature dependence of the relaxivity of water containing Frémy’s salt considering only outer-sphere relaxation as reported previously [8, 15]. The distance of closest approach was *d* = 2.9 Å and the room temperature diffusion coefficient for the Frémy’s salt–water system *D* (298 K) = 2.86 × 10^−5^ cm^2^ s^−1^ [8]. The Stokes–Einstein equation was used to determine *D* as a function of temperature *T* [15], so that $D(T) = D(298\,{\text{K}})\cdot T\cdot \eta (298\,{\text{K}})/(\eta (T)\cdot 298\,{\text{K}})$, with viscosity *η* from Ref. [23]. In addition, the temperature dependence of the diamagnetic relaxation rate in pure water *R*_dia_ was calculated according to $R_{\text{dia}} (T) \sim \tau_{D} (T) \sim 1/D_{\text{water}} (T)$ [24] with *D*_water_(*T*) from the Stokes–Einstein equation and *D*_water_ (298 K) = 2.4 × 10^−5^ cm^2^ s^−1^ [15]. Then, the dependence of the nuclear relaxation time *T*_1n_ on temperature could be estimated from the paramagnetic and diamagnetic relaxation rates:

$$ 1/T_{{1{\text{n}}}} (T) = R_{{1{\text{n}}}} (T) = R_{\text{para}} (T) + R_{\text{dia}} (T) = R(T) \cdot c + R_{\text{dia}} (T). $$

Noting that the heating effect in our experiments should not be concentration-dependent, we find that the observed build-up times at the different concentrations are consistent with *T*_1n_ values in a temperature range of Δ*T* of 25 ± 10 °C. This value is compatible with our former estimate of Δ*T* ≈ 15 K deduced from the cavity quality factor [7]. We note that the DNP build-up described by a monoexponential function is an approximation, if temperature changes during the DNP experiment, yielding an additional time dependence, i.e. *T*_1n_(*t*). Such description is equivalent to the assumption that an average temperature is quickly reached, which is supported by the observations of Kryukov et al. [12]. This model seems to be valid in the present case, because the overall temperature effect as well as the sample volume are very small.

#### Saturation Factors and Evaluation of the OE-DNP at 3.4 T

**Fig. 3:**
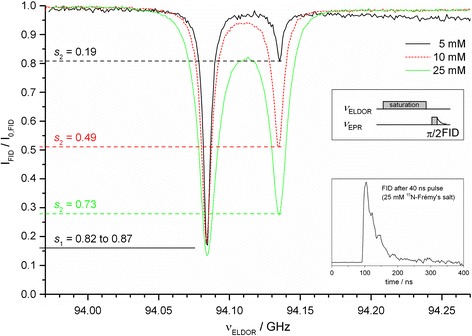
Normalized and baseline corrected EPR FID intensity of the hyperfine line at 94.08 GHz as a function of the ELDOR frequency, i.e. the frequency of the saturating pulse, and of different concentrations of ^15^N-labelled Frémy’s salt in aqueous solution at room temperature. The saturation levels of the individual EPR lines are indicated (the small deviations in *s*_1_ for different samples are attributed to slightly altered coupling conditions in the microwave cavity leading to different quality factors and microwave *B*_1_ field strengths at the sample position). *Inset*: with Frémy’s salt, the EPR FID is observable at 25 mM radical concentration

To rationalize our enhancements in terms of the Overhauser Eq. (2), we have first measured the saturation factors at W-band via a pulsed ELDOR experiment displayed in Fig. 3. So far this type of experiment has not been possible at 94 GHz as the typical EPR FID of TEMPO-derived nitroxides such as TEMPONE-D,^15^N in our previous 94 GHz study [7] decays too quickly to be observed, especially at high polarizer concentrations of 25 mM. However, the use of Frémy’s salt, which exhibits much narrower EPR lines, has enabled us to record the EPR FID intensity (Fig. 3, inset) and thus determine its dependence on the saturation pulse at all three polarizer concentrations. The effective saturation factor *s*_eff_ could be extracted directly from the reduction of each EPR line as $s_{\text{eff}} = (s_{1} + s_{2} )/2$ (Fig. 3) and ranges from 0.5 at 5 mM polarizer concentration to 0.8 at 25 mM (Table 2). Figure 3 shows that the pumped EPR line (at 94.08 GHz) reaches almost complete saturation, i.e. *s*_1_ = 0.82–0.87. Therefore, theoretical maximum enhancements at higher microwave *B*_1_ (but while maintaining the same sample temperature) of ^15^N-Frémy’s salt in water should exceed our experimental values by no more than 15–20 %.

In addition, we calculate the leakage factor $f = \rho /(\rho + w^{0} ) = 1 - R_{\text{dia}} /R_{{1{\text{n}}}}$ for the three samples at an average temperature of *T* = 323 K (Table 2). From these values we derive a DNP coupling factor *ξ* of 0.11–0.12 at 3.4 T and 323 K independent of concentration using the Overhauser Eq. (2). Previous studies employing different methods have predicted coupling factors of 0.11–0.14 at 318–328 K (NMRD) [15] or 0.10–0.12 in the same temperature range (extrapolated from DNP) [12] as well as 0.08 at 318 K (MD) [11]. Therefore, we conclude that the enhancements observed with our W-band DNP setup are well described by the Overhauser mechanism and are subject only to moderate microwave heating, while substantially higher enhancements can only be obtained at higher temperatures.
